# Synthesis, Photoluminescent Characteristics and Eu^3+^-Induced Phase Transitions in Sr_3_Zr_2_O_7_:Eu^3+^ Red Phosphors

**DOI:** 10.3390/nano13091446

**Published:** 2023-04-24

**Authors:** Nianmin Chen, Yunjian Wang, Longfeng Li, Lei Geng, Maolin Zhang

**Affiliations:** 1 Key Laboratory of Green and Precise Synthetic Chemistry and Applications, Ministry of Education, School of Chemistry and Material Science, Huaibei Normal University, Huaibei 235000, China; 12111040679@chnu.edu.cn (N.C.); lilongfeng@chnu.edu.cn (L.L.); 2 School of Materials and Chemical Engineering, Bengbu University, Bengbu 233030, China; 3 College of Physics and Physical Engineering, Qufu Normal University, Qufu 273165, China

**Keywords:** Sr_3_Zr_2_O_7_, Eu^3+^ doped, phosphors, energy transfer

## Abstract

Designing phosphors that are excited by blue light is extraordinarily important for white light-emitting diodes (w-LEDs). In the present study, a new Ruddlesden–Popper type of SZO:*x*Eu^3+^ (*x* = 0.01~0.10) phosphors was developed using solid-state reactions. Interestingly, a Eu^3+^ doping-induced phase transformation from the Sr_3_Zr_2_O_7_ (cubic) to the SrZrO_3_ (orthorhombic) phase was observed, and the impact of the occupied sites of Eu^3+^ ions on the lifetime of Sr_3_Zr_2_O_7_:*x*Eu^3+^ phosphors is discussed in detail. Diffuse reflectance spectroscopy results showed that the band gap of SZO:*x*Eu^3+^ phosphors gradually increased from 3.48 eV for undoped Sr_3_Zr_2_O_7_ hosts to 3.67 eV for SZO:0.10Eu^3+^ samples. The fluorescence excitation spectrum showed that ultraviolet (300 nm), near-ultraviolet (396 nm) and blue light (464 nm) were all effective excitation pump sources of Sr_3_Zr_2_O_7_:*x*Eu^3+^ phosphors, and the strongest emission at 615 nm originated from an electric dipole transition (^5^D_0_→^7^F_2_). CIE coordinates moved from orange (0.5969, 0.4267) to the reddish-orange region (0.6155, 0.3827), and the color purity also increased. The fabricated w-LED was placed on a 460 nm chip with a mixture of YAG:Ce^3+^ and SZO:0.1Eu^3+^ samples and showed “warm” white light with a color rendering index (CRI) of 81.8 and a correlation color temperature (CCT) of 5386 K, indicating great potential for application in blue chip white LEDs.

## 1. Introduction

To achieve the concepts of “energy saving and environmental protection”, plenty of work has been performed on the design and synthesis of phosphors [1]. Compared with traditional lighting fixtures, w-LEDs exhibit more excellent characteristics, such as green environmental protection, high luminous efficiency and long lifetimes [2]. To date, commercial w-LEDs are fabricated by blue light InGaN LED chips with yellow light from Y_3_Al_5_O_12_:Ce^3+^ (YAG:Ce^3+^) phosphors [3,4]. Nevertheless, because of a lack of red light, w-LEDs have the drawbacks of low color rendering indices (CRI < 80%) and high related color temperatures (CCT > 4500 K) [5,6,7]. To conquer this inadequacy, a new technique to fabricate w-LEDs has been proposed to introduce additional red phosphors to blue chips. Compared with blue and green phosphors, the physical and chemical properties of red phosphors are unstable. Accordingly, the development of new and stable red phosphors is still significant [8,9].

Eu^3+^ ions are recognized as red-luminescent activators owing to their ^5^D_0_→^7^F_J_ (J = 1, 2, 3, 4) transitions, and Eu^3+^ has strong and narrow emission at 610–620 nm, so it can be used as an activator of red phosphors [10,11]. The luminous components of red phosphors can be classified into two kinds: banded emission and linear emission. The linear emission of Eu^3+^ ions has greater advantages due to its narrower emission peak, which is helpful to achieve a warm white light output with a high color rendering index. Therefore, selecting Eu^3+^ as an active ion is an ideal choice. However, so far, Eu^3+^-based red phosphors that are excited by blue light are still scarce due to their narrow light absorption cross sections, particularly in the blue spectral range [12]. Accordingly, it is essential to explore Eu^3+^-based red luminescent oxide phosphors excited by blue light. With the development of lighting display materials, various fluorescent materials activated by Eu^3+^ ions have received a lot of attention. In the process of developing these materials, the selection of matrix materials has a great impact on the luminous intensity. Consequently, it is crucial to find a suitable host lattice. For the past few years, lots of work has been performed on zirconate phosphors as hosts for fluorescence centers, as a result of their outstanding chemical and thermal stability and good defect adaptability [13,14,15,16]. Among these compounds, there are enormous amounts of research that have extensively investigated the rare earth (Re = Y, La, Gd) zirconate Re_2_Zr_2_O_7_ ternary oxides [17,18,19], but research on the rare-earth-free earth zirconate oxide phosphors is still limited.

In this work, we report on blue-light-excitable Eu^3+^-doped Sr_3_Zr_2_O_7_ phosphors in hybrid w-LED applications for the first time. A series of Eu^3+^-activated Sr_3_Zr_2_O_7_ phosphors was prepared by solid-state reactions. The structures and luminescent performances of the prepared samples were systematically investigated. According to the XRD measurements, it can be determined that the doping of Eu^3+^ ions can produce phase transitions from the Sr_3_Zr_2_O_7_ (cubic) to the SrZrO_3_ (orthorhombic) phase. The fabricated w-LED, placed on a 460 nm chip with a mixture of YAG:Ce^3+^ and SZO:0.1Eu^3+^ samples, was successfully prepared, and its optical properties were analyzed. This study may provide a novel aspect for the development of fluorescent materials for blue-light-excitable w-LEDs.

## 2. Materials and Methods

SZO:*x*Eu^3+^ samples were synthesized by the solid-state reaction method. Analytically pure SrCO_3_, ZrO_2_ and high-purity reagent Eu_2_O_3_ (4N) were purchased from Sinopharm Chemical Reagent Co., Shanghai, China, and used as received, without purification. During the synthesis process of SZO samples, stoichiometric SrCO_3_ (0.8858 g, 6 mmol) and ZrO_2_ (0.4929 g, 4 mmol) were weighed and ground thoroughly in an agate mortar. The fully mixed powder was put into the programmable high-temperature muffle furnace (STM-8-17) and sintered at 800 °C for 6 h in air. The obtained white powder was ground and pressed into a piece again. Then the piece was sintered at 1500 °C for 8 h. Finally, after the autoclaves were naturally cooled, a white powder product was obtained and then thoroughly ground into fine powder. In compliance with their respective stoichiometric ratios, the doped SZO:*x*Eu^3+^ samples, with doping concentrations of *x* = 0.01, 0.02, 0.04, 0.06, 0.08 and 0.10, respectively, were also synthesized by the same method. The chemical equation used to obtain SZO:*x*Eu^3+^ is as follows:

3(1 − *x*) SrCO_3_ + 2ZrO_2_ + 3*x*/2Eu_2_O_3_→Sr_3(1−*x*)_Zr_2_O_7_:*x*Eu^3+^

### 2.1. Fabrication of Warm w-LED Devices

For comparison, two kinds of w-LED devices were fabricated based on the prepared SZO:0.10Eu^3+^ red phosphor. Commercial blue phosphor BaMgAl_10_O_17_:Eu^2+^, green phosphor (Ba,Sr)_2_SiO_4_:Eu^2+^ (Yantai Shield, Yantai, China) and yellow phosphor Y_3_Al_5_O_12_:Ce^3+^ (YAG:Ce) (Shenzhen Looking Long Technology Co., Ltd., Shenzhen, China) were used with 400 nm and 465 nm chips (San’an Optoelectronics Co., Ltd., Sanya, China) to fabricate the warm w-LED devices. The first LED (denoted as LED1) was fabricated using the prepared SZO:0.10Eu^3+^ red phosphor, (Ba,Sr)_2_SiO_4_:Eu^2+^ commercial green phosphor and BaMgAl_10_O_17_:Eu^2+^ (BAM:Eu^2+^) blue phosphor on the surface of a 400 nm near-UV LED chip with a R/G/B phosphor mass of 6:2:1. Another white LED lamp (denoted as LED2) was fabricated by using a 465 nm blue LED chip coated with a mixture of the commercial yellow phosphor Y_3_Al_5_O_12_:Ce^3+^ (YAG:Ce) and the prepared SZO:0.10Eu^3+^ red phosphor with a mass ratio of 1:4. Finally, the fabricated devices were dried at 100 °C for 2 h in the oven, and, subsequently, electroluminescence measurements were carried out at drive currents varying from 20 mA to 300 mA on an integrating sphere spectroradiometer system.

### 2.2. Phosphor Characterization

The phases and crystal microstructures of the prepared samples were identified by the Bruker D8 Advance X-ray powder diffractometer (Bruker Corporation, German). The lattice parameters of the SZO:*x*Eu^3+^ samples were refined by the Rietveld refinement technique, based on general structure analysis system (GSAS1) software (A.C. Larson, R.B. Von Dreele, NM, USA) [20,21]. The surface morphologies and elemental analyses of the phosphors were documented on a FEIS irion200 scanning electron microscope (Carl Zeiss AG, Oberkochen, German) working at conditions of 5 kV and 10 mA. Infrared spectra were obtained on a Nicolet iS50 FT-IR spectrophotometer (Thermo Scientific Nicolet iS50, Waltham, MA, USA) by the KBr pellet technique. UV-Vis absorption spectra of the samples were documented using a UV2600 UV-Vis spectrophotometer (Shimadzu Corporation, JPN) with barium sulphate as a reference in a range of 200–800 nm. A photoluminescence spectrum was obtained using an F97 Pro fluorescence spectrophotometer (Shanghai Prism Technology Co., Ltd. Shanghai, China) at room temperature. The fluorescence attenuation curve was measured by a Hitachi F-7000 fluorescence spectrophotometer (Hitachi Production Co., Ltd., Tokyo, Japan). In order to ensure the accuracy of experimental data, some experimental conditions, such as slit width, sample mass and strength of illumination, were kept constant. Color coordinates and color temperature were calculated by CIE1931.

## 3. Results and Discussion

**Figure 1:**
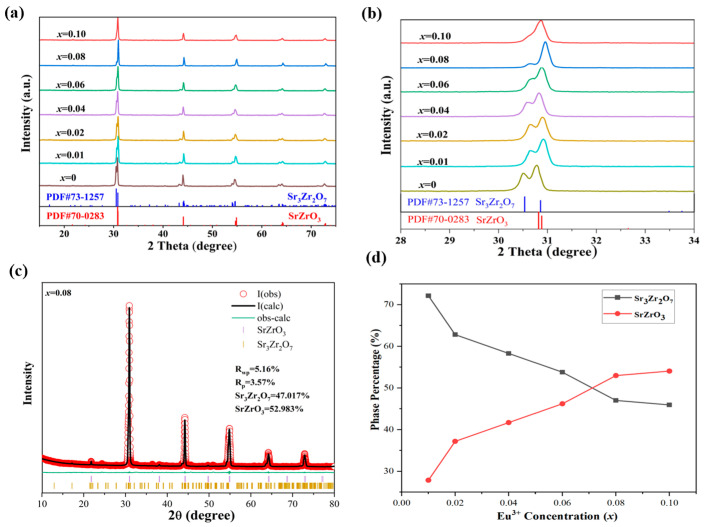
(**a**) XRD and (**b**) enlarged XRD patterns in the given range of the Sr_3_Zr_2_O_7_ and SZO:*x*Eu^3+^ (*x* = 0.01–0.10) samples. (**c**) The refinement profiles of SZO:0.08Eu^3+^. (**d**) The dependence of the phase percentage of Sr_3_Zr_2_O_7_ and SrZrO_3_ on the doping concentration of Eu^3+^.

The phase purity and structural evolutions of the synthesized SZO:*x*Eu^3+^ phosphors were detected by XRD analysis. The full range of XRD patterns of Sr_3_Zr_2_O_7_ and SZO:*x*Eu^3+^ (*x* = 0.01–0.10) phosphors are displayed in Figure 1a. It can be deduced from XRD analysis that peaks of the undoped sample are in accord with the standard ones of Sr_3_Zr_2_O_7_ (JCPDS card No. 73–1257). As shown in the enlarged XRD patterns in Figure 1b, the undoped phosphor was single-phase without any impurities, and when the concentration of Eu^3+^ increased from 0.01 to 0.10, the strongest diffraction peak at 30.53° gradually shifted in the direction of the large angle, while the intensity of the peak gradually weakened until the Eu^3+^ doping concentration reached 0.10 and the peak almost disappeared; the peak at 30.86° can be attributed to SrZrO_3_ (JCPDS card No. 70-0283), which indicates that the doping of Eu^3+^ is conducive to the transformation from cubic Sr_3_Zr_2_O_7_ to an orthorhombic SrZrO_3_ phase. To further clarify the proportions of the phase transition, the XRD refinement technique was used to calculate the percentages of Sr_3_Zr_2_O_7_ and SrZrO_3_ at different Eu^3+^ ion doping concentrations. As shown in Figure 1c, the refined results are in good agreement with the experimental results. Figure 1d shows the correlation between the Eu^3+^ doping concentration and the relative content of the two phases. It is noted that the phase content of Sr_3_Zr_2_O_7_ decreased, while that of SrZrO_3_ increased as the doping concentration increased.

**Figure 2:**
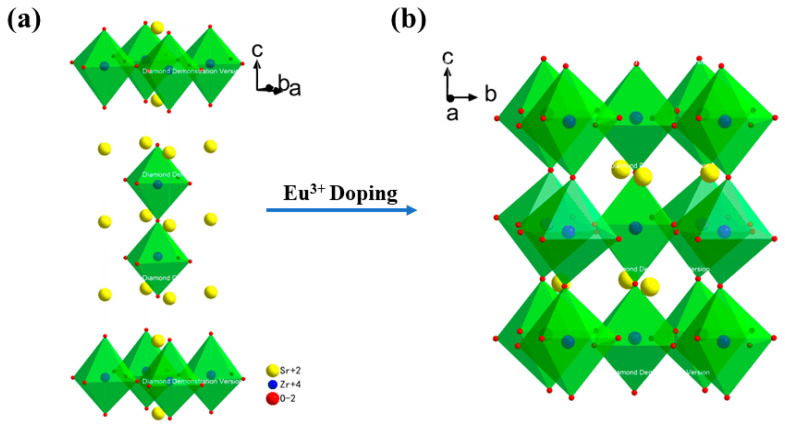
(**a**) Crystal structure of Sr_3_Zr_2_O_7_ and (**b**) crystal structure of SrZrO_3_. (a, b, c present crystallographic coordinate systems).

As can be seen from Figure 2a,b, both Sr_3_Zr_2_O_7_ and SrZrO_3_ belong to the same Ruddlesden–Popper family with a general A_n+1_B_n_O_3n+1_ (n = 1, 2, 3) formula, but in different space groups: Sr_3_Zr_2_O_7_ (*Pmmm*) and SrZrO_3_ (*Pbnm*) [22,23]. The Sr_3_Zr_2_O_7_ crystal is a three-dimensional structure composed of ZrO_6_ octahedron, and the ZrO_6_ octahedron is linked at the corner. The Zr atoms had the same coordination number in both the Sr_3_Zr_2_O_7_ and the SrZrO_3_, while there were different ZrO_6_ octahedron stacking patterns. Different kinds of Sr^2+^ sites existed in the Sr_3_Zr_2_O_7_ crystal. One kind of Sr^2+^ atoms was bonded to twelve O^2-^ atoms to form SrO_12_ cuboctahedra. The second kind of Sr^2+^ atoms was linked to nine O^2-^ atoms with Sr–O bond distances ranging from 2.40 to 2.98 Å. The crystal radii of nine- and twelve-coordinated Sr^2+^ ions were 1.31 Å and 1.58 Å, respectively; while the radius of six-coordinated Zr^4+^ was 0.86 Å in the crystal [24]. The Sr_3_Zr_2_O_7_ host supplied two probable occupied sites (Sr^2+^ or Zr^4+^) for Eu^3+^ ions. The radii of the doped Eu^3+^ ions varied from 1.08 (CN = 6) to 1.26 Å (CN = 9) in different coordination environments. From the angle of the atomic radius, was is generally concluded that the doped Eu^3+^ (1.26 Å, CN = 9) tended to substitute for the larger Sr^2+^ (1.31 Å, CN = 9) sites. On one hand, the substitution of Eu^3+^ for Sr^2+^ resulted in a lattice contraction, as indicated by the high angle shift of the diffraction peak in Figure 1b. On the other hand, this kind of substitution also led to a lattice distortion due to the mismatch of the ion radius and charge. As mentioned above, another potential site for Eu^3+^ is Zr^4+^. The substitution of Eu^3+^ for Zr^4+^ just compensates for the charge balance. Therefore, it is rational to draw a conclusion that the doped Eu^3+^ ions occupied both the Sr^2+^ and the Zr^4+^ sites in the Sr_3_Zr_2_O_7_ at high doping levels. Orthorhombic Sr_3_Zr_2_O_7_ is a metastable structure, and phase transitions occur easily under high temperatures. This work finds that the transformation from Sr_3_Zr_2_O_7_ to SrZrO_3_ also occurs along with the increase in Eu^3+^.

**Figure 3:**
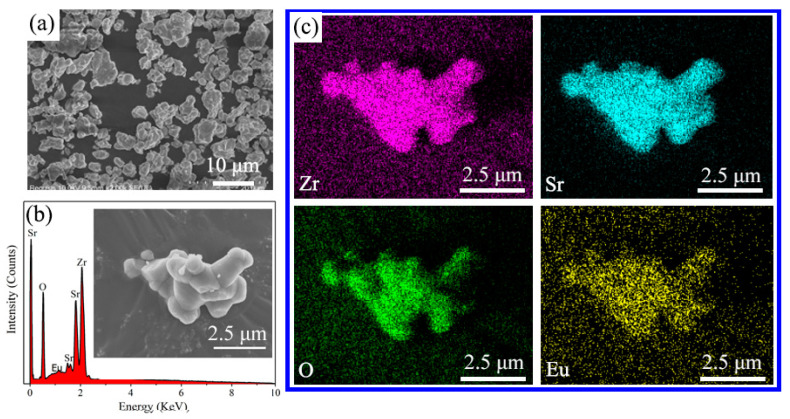
(**a**) SEM images, (**b**) EDS images and (**c**) elemental mappings of a selected SZO:0.10Eu^3+^ single particle at a 2.5 μm scale.

The morphologies and element distributions of the SZO:0.10Eu^3+^ phosphors were tested using energy dispersive spectroscopy (EDS) and scanning electron microscopy (SEM). As shown in Figure 3a, the phosphors are basically composed of particles with different shapes and sizes, with an average size of about 10 μm. The energy dispersive spectra in Figure 3b demonstrate that the expected elements of Sr, Zr, O and Eu all existed in the SZO:*x*Eu^3+^ phosphor, and no other additional peaks were observed. The element mapping images are given in Figure 3c, and it is found that all elements were uniformly dispersed in the SZO host.

**Figure 4:**
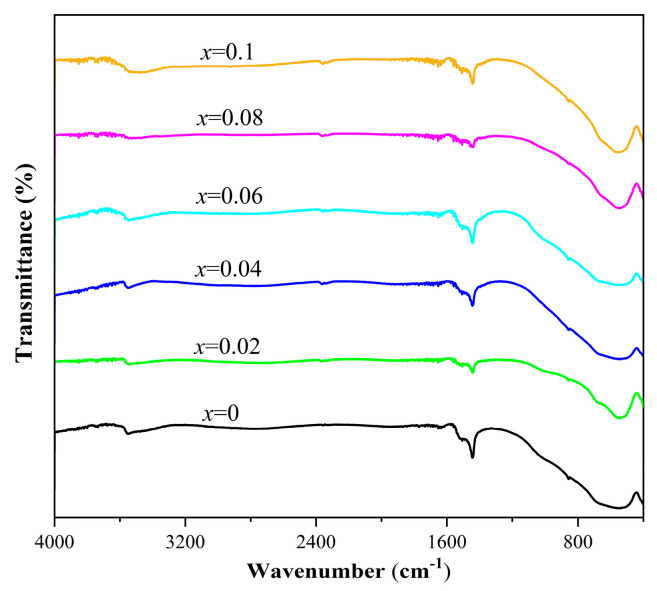
FT−IR spectra of the Sr_3_Zr_2_O_7_ and SZO:*x*Eu^3+^ (*x* = 0.02–0.10) phosphors.

Eu^3+^ doping influences on the chemical bond of Sr_3_Zr_2_O_7_ and SZO:*x*Eu^3+^ (*x* = 0.02–0.10) samples were further studied via infrared spectroscopy. As Figure 4 shows, in the range of 4000–400 cm^−1^, all Sr_3_Zr_2_O_7_ and SZO:*x*Eu^3+^ phosphors displayed similar spectra. The broad band at 544.8 cm^−1^ corresponds to the stretching vibrations of the Zr-O band in the ZrO_6_ octahedron [25]. The band at 3556 cm^−1^ corresponds to the O-H stretching vibrations of surface-absorbed water molecules, and the peak at 2360 cm^−1^ is assigned to the CO_2_ molecule in air [26].

**Figure 5:**
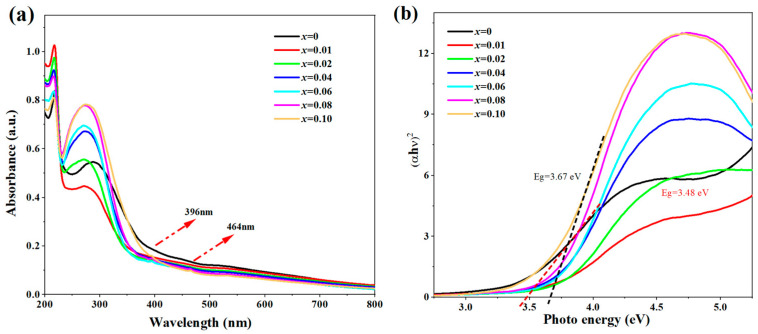
(**a**) UV-Vis spectra and (**b**) a plot of (*αhν*)^2^ vs. *hν* of the prepared Sr_3_Zr_2_O_7_ and SZO:*x*Eu^3+^ (*x* = 0.01–0.10) samples.

The optical properties of luminescent materials are usually related to the band structure, and the UV-Vis absorption spectra of the solid samples were measured on a UV2600 UV-Vis spectrophotometer connected with an integrating sphere. As shown in Figure 5a, the strong and sharp absorption located at 380–250 nm belongs to the inter-band absorption of the SZO host. Two minor bands were observed at 396 and 464 nm for the higher Eu^3+^-doped SZO:*x*Eu^3+^ samples, and they became stronger with the increase of the doping content, which stemmed from the spin and parity-forbidden 4f-4f transition of the Eu^3+^ ions [27]. The bandgap *E_g_* of the SZO:*x*Eu^3+^ samples was obtained via the Tauc formula [28]:

[*F*(*R_∞_*) *hv*]^1/n^ = *A* (*hv* − *E_g_*) (1)

in which *A* is the characteristic constant of semiconductors, *hv* is the incident photon energy, *E_g_* is the band gap energy, n is a constant that determines the transition nature of a semiconductor (n = 1/2 for a direct transition or 2 for an indirect transition) [29] and *F*(*R_∞_*) is the Kubelka–Munk function, which can be defined as [30]:

*F*(*R_∞_*) = (1 − *R*)^2^/2*R* = *K*/*S* (2)

where *R* is the diffuse reflectance, and *K* and *S* are the Kubelka–Munk absorption and scattering coefficients, respectively. The [*F*(*R**_∞_***)*hv*]^2^ vs. *hv* plots are displayed in Figure 5b. The band gap *E_g_* for the undoped Sr_3_Zr_2_O_7_ host was determined about 3.48 eV, and it gradually increased to 3.67 eV for the SZO:0.10Eu^3+^ sample, which is exceedingly close to the 3.63 eV of SrZrO_3_ reported in the literature [31]. In addition, doping Eu^3+^ ions may cause lattice distortion and widen the bandgap of the material, and structural changes within the material can affect the position of the absorption band [32,33]. All of these results further demonstrate that Eu^3+^ doping leads to the transition from Sr_3_Zr_2_O_7_ to SrZrO_3_.

**Figure 6:**
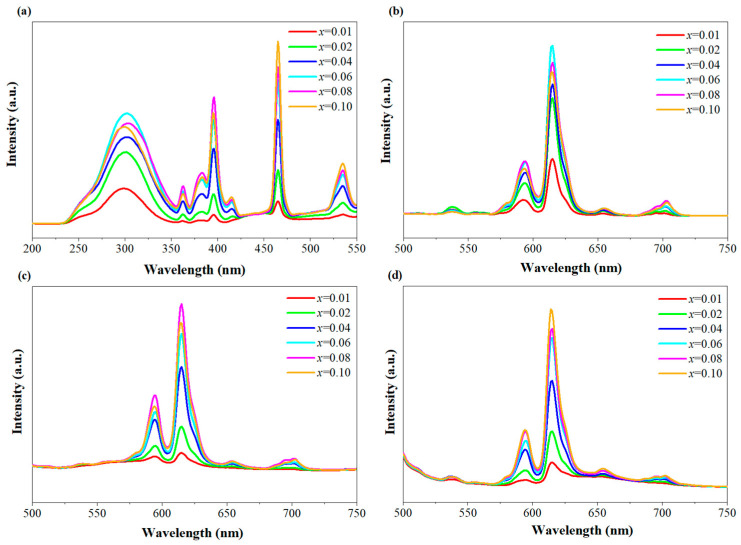
(**a**) PLE spectra of SZO:*x*Eu^3+^ monitored at 615 nm. (**b**–**d**) PL spectra of SZO:*x*Eu^3+^ under 300, 396 and 464 nm excitations.

PLE spectra of SZO:*x*Eu^3+^ phosphors monitored at 615 nm are present at Figure 6a. The excitation spectra of all samples were analogous in shape. The peak from 250 nm to 350 nm is attributed to the charge transfer (CT) transition from O 2p to Eu 4f orbital. It is worth observing that the CT band remained almost unchanged in position, while the intensity of the CT band increased significantly with the increasing of the Eu^3+^ content, which demonstrates that Eu^3+^ doping can improve the energy transfer efficiency between a SZO matrix and Eu^3+^ ions. The signals from Eu^3+^ 4f-4f transitions were present at 361 nm (^7^F_0_→^5^D_4_), 381 nm (^7^F_0_→^5^L_7_), 395 nm (^7^F_0_→^5^L_6_), 414 nm (^7^F_0_→^5^D_3_) and (^7^F_0_→^5^D_1_) [34,35], respectively. Moreover, the strongest band among them was observed at 464 nm and was originated from the electronic transition. It is rewarding to point out that the strongest excitation peak was located at 464 nm. This indicates that the phosphor has excellent blue light conversion ability. The three strongest excitation bands for the SZO:*x*Eu^3+^ phosphor were found at 300 nm (CTB), 396 nm and 464 nm, which indicates that UV, near-UV and, especially, blue light are all effectual pumping sources to generate Eu^3+^ emissions. Wavelengths of 300, 396, and 464 nm were selected to obtain the emission spectra. Figure 6b–d shows the emission spectra of all SZO:*x*Eu^3+^ phosphors under 300, 396, and 464 nm excitations, respectively. It was found that all SZO:*x*Eu^3+^ phosphors showed very similar emissions, except for their intensity, under different excitations. One peak at 537 nm was originated from the transition of Eu^3+^ from ^5^D_1_ to ^7^F_1_, and another five peaks were observed at 578 (very weak), 593, 615, 654 and 703 nm and originated from the radiative ^5^D_0_→^7^F_J_ transitions (J = 0–4) [36,37]. The strongest emission at 615 nm is attributed to the electric dipole ^5^D_0_→^7^F_2_ transition of Eu^3+^, and another emission at 593 nm is ascribed to the magnetic dipole ^5^D_0_→^7^F_1_ transition. The excellent luminescence under UV light indicates an effective energy transfer from the SZO host to the doped Eu^3+^ ions. The concentration-quenching phenomena were firstly observed at 6% for SZO:Eu^3+^ phosphors under an excitation of 300 nm, while it reached 8% under an excitation of 396 nm. Different fluorescence-quenching concentrations observed under different excitation conditions may result from the fact that either the nonradiative transition efficiency from the conduction band, ^5^H_6_, and the ^5^D_2_ level to ^5^D_0_ level or the radiative transition (^5^D_0_→^7^F_2_) was affected by the Eu^3+^ doping concentration. Both the high PL intensity and the high quenching concentration under blue light excitation (464 nm) further indicate that the prepared SZO:*x*Eu^3+^ phosphors have great potential for application in blue-chip w-LEDs.

It is generally believed that there are two kinds of non-radiation concentration-quenching mechanisms in fluorescent powder: exchange interactions or electric multipolar interactions. For the purpose of identifying the concentration-quenching mold of the SZO:*x*Eu^3+^ samples, a commonly used parameter, the critical distance (Rc), can determine the specific type of concentration quenching, and its value is calculated by the Blasse formula [38]:

*R_c_* = 2[3*V*/(4*x_c_ N*)]^1/3^ (3)

in which *V* is the lattice volume, *N* is cell formula units and *x*_c_ is the optimum concentration of the dopant. For the SZO:*x*Eu^3+^ samples, *V* = 275.81 Å^3^, *N* = 4 and *x*_c_ = 10% under blue excitation, and the obtained critical distance (10.96 Å) was larger than 5 Å [39,40]. Hence, the multi-dipole interaction was the major cause for concentration quenching in the SZO:*x*Eu^3+^ phosphors.

**Figure 7:**
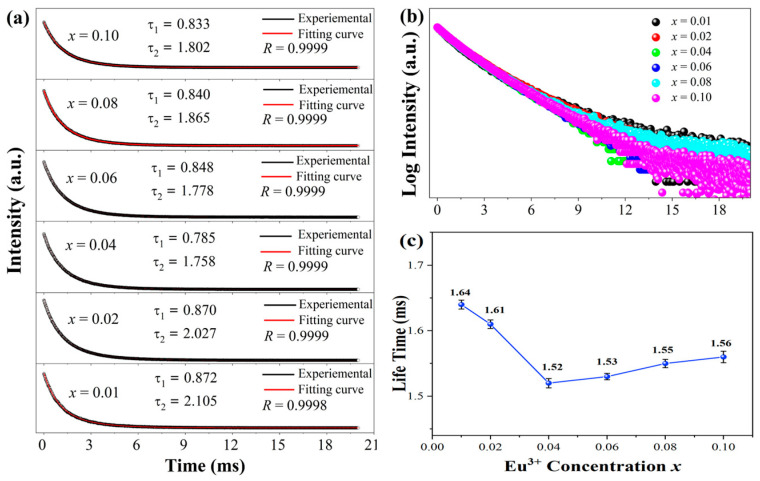
(**a**,**b**) The decay and the fitting curves and (**c**) the lifetimes of the SZO:*x*Eu^3+^ (*x* = 0.01–0.10) phosphors for 615 nm (^5^D_0_→^7^F_2_) emissions excited at 465 nm.

The lifetime of Eu^3+^ ions usually varied in different hosts, and the lifetime of the prepared SZO:*x*Eu^3+^ phosphor was determined through a time-domain method. The PL decay curves of SZO:*x*Eu^3+^ phosphors investigated by monitoring the ^5^D_0_→^7^F_2_ emissions (615 nm) are shown in Figure 7. The luminescence decay curves for all of the SZO:*x*Eu^3+^ samples were well-fitted by the double exponential equation [41]:

*I_t_* = *I*_0_ + *A*_1_ exp(−*t*/*τ_1_*) + *A*_2_ exp(−*t*/*τ_2_*) (4)

where *I_t_* represents the luminescence intensity at time “*t*”, *I*_0_ depicts the background intensity, *A*_1_ and *A*_2_ refer to the exponential coefficient of the functional model, *τ*_1_ and *τ*_2_ are the decay times for the exponential components and *t* is the time. The double exponential fitting of the fluorescence lifetime is consistent with the *A/B*-double occupation of Eu^3+^ ions in the SZO matrix. The corresponding *τ*_1_ and *τ*_2_ for all SZO:*x*Eu^3+^ samples are shown in Figure 7a, and the average decay time *τ* was calculated using the formula below [42]:

*τ* = (*A*_1_*τ*_1_^2^ + *A*_2_*τ*_2_^2^)/(*A*_1_*τ*_1_ + *A*_2_*τ*_2_) (5)

The calculated average lifetimes of SZO:*x*Eu^3+^ phosphors are illustrated in Figure 7c. It was found that the fluorescence lifetime *τ* decreased from 1.64 ms to 1.52 ms with the increase of the Eu^3+^ doping content from 0.01 to 0.04. However, it was different from the monotonous decrease in the fluorescence lifetime of general phosphors; *τ* shows a slight increase from 1.52 ms to 1.56 ms when the doping concentration further increased from 0.04 to 0.10. It is well known that the change in lifetime is related to the non-radiative transition between the luminous centers [43], and we infer that Eu^3+^ ions replaced a small amount of Zr^4+^ ions at a higher content of Eu^3+^ ions, which effectively weakened the non-radiative energy transfer process between Eu^3+^. Therefore, it can be further proven that the doped Eu^3+^ ions occupied both Sr^2+^ and Zr^4+^ sites in the Sr_3_Zr_2_O_7_ at high doping levels.

**Figure 8:**
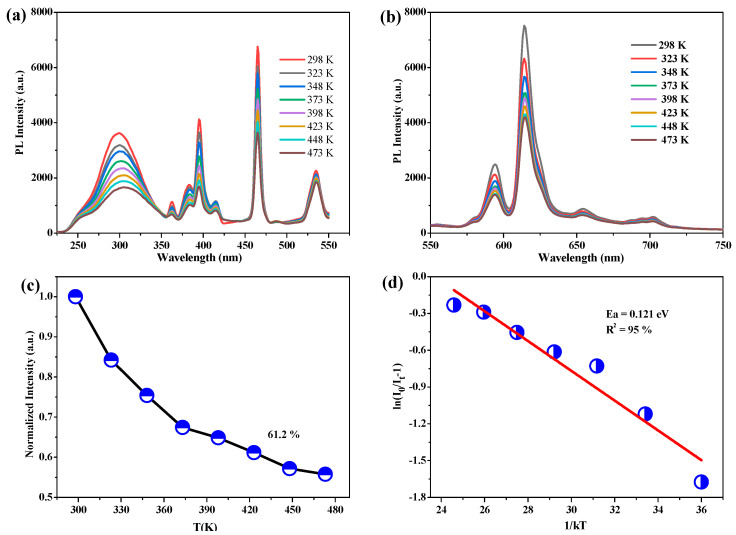
(**a**,**b**) Temperature−dependent PLE and PL spectra of SZO:0.1Eu^3+^, (**c**) relative PL intensities and (**d**) plot of ln(*I*_0_/*I_T_* − 1) vs. 1/*kT* under a 464 nm excitation.

The increase in in situ working temperature had an adverse impact on the luminescent properties of the phosphor; thus for the purpose of improving the practical efficiency of phosphors, the high thermal stability of phosphors is particularly valuable. Figure 8a shows the PLE spectra of the SZO:0.1Eu^3+^ phosphor monitored at 615 nm under diverse temperatures in the scope of 298–473 K. Apparently, due to the existence of the thermal quenching effect, the working efficiency of phosphors will decline with the increasing of the working temperature. The PL spectra of SZO:0.1Eu^3+^ monitored at 464 nm under various temperatures in the scope of 298–473 K is shown in Figure 8b. Similarly, the intensity of all emission peaks weakened with the increasing of the temperature. Moreover, the relative intensity change in the 615 nm emission with the temperature is given in Figure 8c; when the temperature went up to 150 °C [44], the prepared SZO:0.1Eu^3+^ phosphor maintained a 61.5% luminous intensity compared to its room temperature intensity, which is almost similar to K_3_LuSi_2_O_7_:Eu^2+^ phosphor (59%) [45]. Besides temperature, another parameter to assess the degree of the thermal quenching of phosphors is activation energy, which can be estimated using the following equation [46,47]:

*I_T_* = *I*_0_*/*[1 + *Aexp*(−Δ*E*/*kT*)] (6)

The above formula can be transformed into ln(*I*_0_/*I_T_* − 1) = ln*A* − Δ*E*/(*kT*). The ln(*I*_0_/*I_T_* − 1) vs. 1/*kT* curves for the strongest emission of SZO:0.1Eu^3+^ phosphor is displayed in Figure 8d. The activation energy Δ*E* was determined to be about 0.121 eV by the linear fitting of the curve.

**Figure 9:**
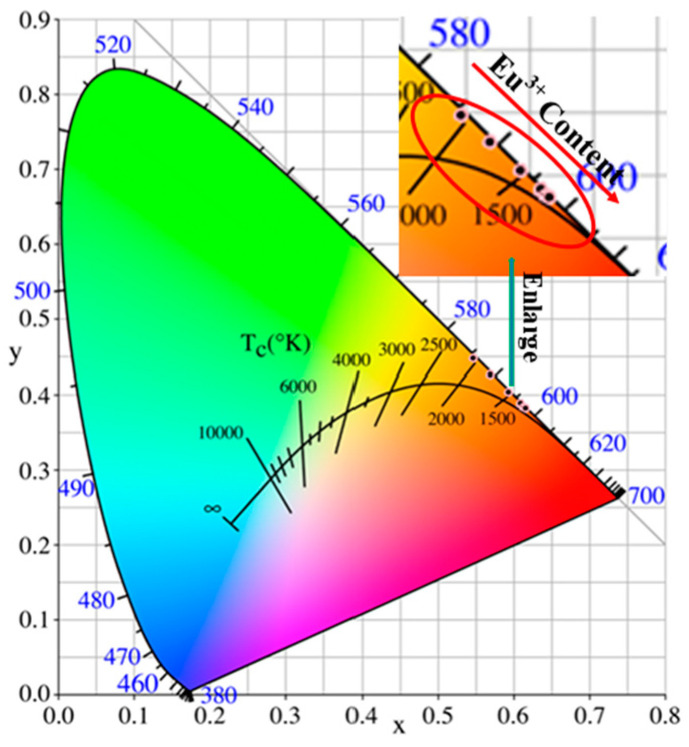
CIE chromaticity coordinates of SZO:*x*Eu^3+^ (*x* = 0.01–0.10) excited at 464 nm.

The color coordinates of the phosphor are a vital parameter in its application. The color coordinates were obtained by CIE1931 software (International Commission on Illumination), according to the luminescence spectrum of the phosphor. The chromaticity coordinates of the prepared SZO:*x*Eu^3+^ phosphors under 464 nm excitation are plotted in Figure 9. The luminescence of the SZO:*x*Eu^3+^ phosphors clearly shifts from orange (0.5969, 0.4267) for 0.02 to the reddish-orange region (0.6155, 0.3827) for the enhancement of Eu^3+^ luminescence with increased Eu^3+^ doping content. The correlated color temperature (*CCT*) was calculated using the Mecamy empirical formula [48]:

*CCT* = −449*n*^3^ + 3525*n*^2^ − 6823.3*n* + 5520.33 (7)

in which *n* = (*x* − *x_e_*)/(*y* − *y_e_*) and (*x_e_*,*y_e_*) is the chromaticity epicenter, which is located at (0.3320, 0.1858). The detailed color coordinates and *CCT* values of all samples are summarized in Table 1. The *CCT* of each sample is located in the range of 1729 to 1796 K. Moreover, color purity (*CP*) is another important parameter to evaluate the performance of phosphors and can be calculated by following equations [49]:

(8) CP=(x−xe)2+(y−ye)2(xd−xe)2+(yd−ye)2

where *n* is equal to (*x* − *x_e_*)/(*y* − *y_e_*); (*x_e_*, *y_e_*) (*x*, *y*), and (*x_d_*, *y_d_*) are the chromaticity epicenter, chromaticity coordinates of SZO:*x*Eu^3+^phosphors and the chromaticity coordinates of the strongest emission of SZO:*x*Eu^3+^ phosphors, respectively. The *CCTs* and *CPs* of all SZO:*x*Eu^3+^ samples are also displayed in Table 1. Apparently, the color purity of SZO:*x*Eu^3+^ phosphors was gradually enhanced from 88.6% to 90.1% when the doping concentration of Eu^3+^ increased from 0.01 to 0.10.

**Figure 10:**
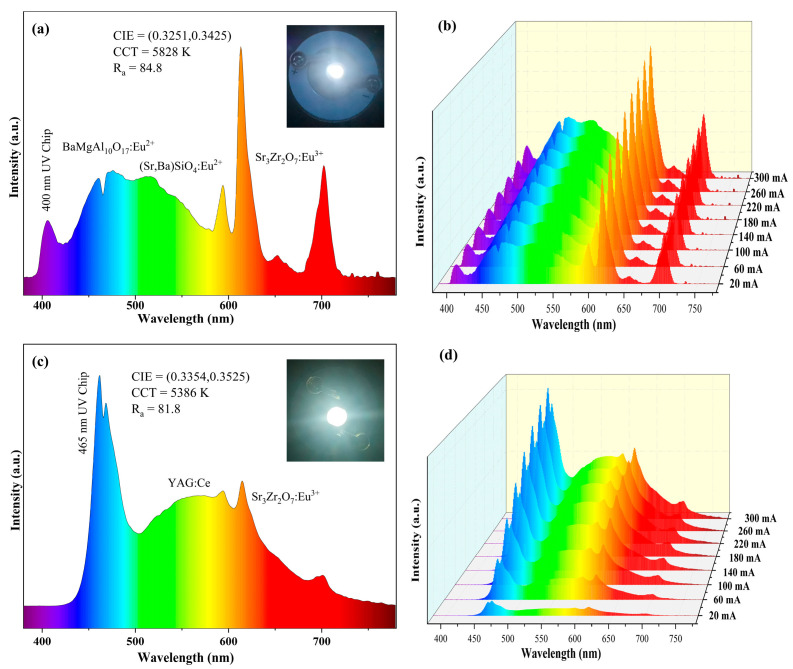
(**a**,**c**) EL spectra of the fabricated LED lamps using 400 and 465 nm LED chips at a 300 mA current. (**b**,**d**) EL spectra of the fabricated LEDs on 400 and 465 nm LED chips driven at various currents.

In order to evaluate the potential application of SZO:0.1Eu^3+^ phosphor in white LEDs, two types of LEDs were fabricated by depositing SZO:0.1Eu^3+^ phosphors on 400 and 465 nm chips. The emission spectra and digital photographs of the two LED devices are depicted in Figure 10. The LED device based on the 400 nm chip showed bright warm white light and had good photoelectric characteristics of the CIE chromaticity coordinates (0.3251, 0.3425), a moderate *CCT* of 5828 K and a high CRI of 84.8. As shown in Figure 10c, the fabricated LED device on the 460 nm chip showed a similar *CIE* chromaticity coordinate (0.3354, 0.3525), a lower *CCT* (5386 K) and slightly smaller *CRI* (81.8). Moreover, it was found that the red emission intensity of the LED device on the 465 nm chip was relatively lower than that on the 400 nm chip, due to the absorption cross section of Eu^3+^ ions at different wave ranges. Figure 10b,d demonstrates the emission spectra of the two fabricated LED lamps driven at diverse currents. It was observed that all emissions gradually increased as the operational currents increased from 20 to 300 mA. These results show that the reported SZO:*x*Eu^3+^ phosphor is expected to be used to manufacture high-power white LEDs.

## 4. Conclusions

Eu^3+^-activated SZO red phosphors with Ruddlesden–Popper structures were synthesized using solid-state reactions. X-ray powder diffraction results showed that Eu^3+^ doping at Sr^2+^ sites induced phase transformations from Sr_3_Zr_2_O_7_ to SrZrO_3_ due to the mixed occupancy at the Sr^2+^ and Zr^4+^ sites. In addition, all the prepared SZO:*x*Eu^3+^ phosphors were able to be effectively excited by ultraviolet, near-ultraviolet and blue light sources. This synthesized phosphor can be well-combined with commercial green and blue phosphors to create bright white light with lower *CCTs* and large *CRIs*. Therefore, the SZO:*x*Eu^3+^ phosphor reported in this work has potential applications in w-LEDs for high-quality lighting and displays.

## Figures and Tables

**Table 1:** CIE coordinates, CCTs and CPs of SZO:*x*Eu^3+^ (*x* = 0.01–0.10) phosphors excited at 464 nm.

Sr_3_Zr_2_O_7_:*x*Eu^3+^	1%	2%	4%	6%	8%	10%
CIE *x*	0.5969	0.5936	0.6099	0.6092	0.6136	0.6155
CIE *y*	0.4267	0.4038	0.388	0.3888	0.3846	0.3827
CCT (K)	1724	1711	1752	1747	1778	1796
CP (%)	88.60	85.54	88.87	88.72	89.68	90.10

## Data Availability

The data in this study are available upon request from the corresponding author.
